# High PD-1 expression level is associated with an unfavorable prognosis in patients with cervical adenocarcinoma

**DOI:** 10.1007/s00404-020-05589-0

**Published:** 2020-05-20

**Authors:** Masako Ishikawa, Kentaro Nakayama, Kohei Nakamura, Hitomi Yamashita, Tomoka Ishibashi, Toshiko Minamoto, Kouji Iida, Sultana Razia, Noriyoshi Ishikawa, Satoru Nakayama, Yoshiro Otsuki, Satoru Kyo

**Affiliations:** 1 Department of Obstetrics and Gynecology, Shimane University Faculty of Medicine, Enyacho 89-1, Izumo, Shimane 6938501 Japan; 2 Department of Organ Pathology, Shimane University Faculty of Medicine, Izumo, 6938501 Japan; 3 Department of Obstetrics and Gynecology, Seirei Hamamatsu Hospital, Hamamatsu, 4308558 Japan; 4 Department of Organ Pathology, Seirei Hamamatsu Hospital, Hamamatsu, 4308558 Japan

**Keywords:** Cervical adenocarcinoma, Immune-checkpoint inhibitor, Programmed cell death-1, Programmed cell death-ligand 1, CD8 expression, Survival analysis, Combination therapy

## Abstract

**Purpose:**

The effectiveness of immunotherapy for cervical adenocarcinoma (CA) has not been demonstrated yet. Programmed cell death 1 (PD-1), programmed cell death-ligand 1 (PD-L1), and CD8 may be used as biomarkers of response to immune therapy in CA patients. In the present study, we aimed to investigate whether the expression levels of PD-1, PD-L1, and CD8 can predict the prognosis of patients with CA and their response to immune checkpoint inhibition therapy.

**Methods:**

In the present study, the clinical stage for all 82 patients with cervical adenocarcinoma was classified according to the guidelines of the International Federation of Gynecology and Obstetrics (FIGO); there were 5, 48, 5, 14, 8, and 2 patients with stage IA, IB, IIA, IIB, IIIB, and IVB disease, respectively. The levels of PD-1, PD-L1, and CD8 were analyzed by the immunohistochemical analysis of the formalin-fixed paraffin-embedded tumor samples. The correlation between the expression levels and patient prognosis was analyzed using the Kaplan–Meier method and univariate and multivariate Cox proportional hazard regression models.

**Results:**

We observed a significant inverse correlation between the expression of PD-1 and CD8 (*p* = 0.001, chi-square test). We also found a significant inverse correlation between the expression of PD-L1 and CD8 (*p* = 0.027). The overall survival and progression-free survival rates were significantly worse in patients with positive PD-1 expression (*p* = 0.031; *p* = 0.087, respectively).

**Conclusion:**

Our results suggest that a high PD-1 expression is associated with a poor prognosis in patients with CA. Further research is necessary to identify the molecular mechanisms that mediate this association.

**Electronic supplementary material:**

The online version of this article (10.1007/s00404-020-05589-0) contains supplementary material, which is available to authorized users.

## Introduction

Cervical cancer is the fourth most common cancer in women according to the World Health Organization. In Japan, there are 13,000 new cases of cervical cancer and 3500 cervical cancer-related deaths every year [1, 2]. Thus, cervical adenocarcinoma has become a major health concern among women because of its high prevalence and poor prognosis. Adenocarcinoma accounts for approximately 10–25% of uterine cervical cancer cases [1–4]. In Japan, cervical adenocarcinoma is one of the primary health problems in young women because the morbidity and mortality associated with it have increased in the past 2 decades [5].

Recently, several studies have reported new immunotherapy options for cancer that effectively stimulate the host immune response to eliminate tumor cells. For example, treatment with nivolumab or pembrolizumab, antibodies against programmed cell death-1 (PD-1), has markedly improved the overall survival of patients suffering from malignant tumors with poor prognoses. However, as these immune checkpoint inhibitors are effective only against a few types of cancers, only a small percentage of cancer patients benefit from their use. Therefore, there is a crucial need to identify potential markers that can predict the success of these new immune therapies. To achieve this goal, many studies have investigated the tumor microenvironment and clarified the mechanisms of immunoediting and tumor immunogenicity, as well as the composition of the tumor-infiltrating cells, such as lymphocytes and macrophages.

Furthermore, several studies have investigated whether PD-1 can be used as a predictive biomarker of disease treatment success or as a predictor of poor prognosis. The effectiveness of immunotherapy alone or as a combination therapy in cervical adenocarcinoma has not been demonstrated yet. Thus, in this study, we aimed to investigate whether the expression levels of PD-L1, PD-1, and CD8 can predict the prognosis of cervical adenocarcinoma patients and their response to immune-checkpoint inhibition therapy.

## Materials and methods

### Tissue samples

Tissue samples were obtained from the Department of Obstetrics and Gynecology at Shimane University School of Medicine (Shimane, Japan) and Seirei Hamamatsu Hospital (Shizuoka, Japan) between 2003 and 2017. A total of 82 samples (obtained during surgery or biopsy) were collected from patients with uterine cervical adenocarcinoma. The diagnoses were confirmed by an expert gynecopathologist in our institution. Most patients were primarily treated with surgery and received adjuvant therapy, such as chemotherapy, radiotherapy, or concurrent chemoradiotherapy (CCRT) with cisplatin (weekly dose of 40 mg/m^2^). Clinical information was obtained, retrospectively, from electronic medical records. The acquisition of tumor tissues was approved by the Shimane University Institutional Review Board (IRB No. 20070305-1 and No. 20070305-2).

### Immunohistochemical analysis

The expression levels of PD-1, PD-L1, and CD8 were evaluated by immunohistochemical analysis (IHC). Formalin-fixed and paraffin-embedded (FFPE) Sects. (4 μm thick) were dewaxed in xylene and hydrated in graded alcohol solutions. After antigen retrieval in a sodium citrate buffer, the slides were incubated overnight at 4 °C with antibodies against CD-8 (1:100; Roche, Basel, Switzerland), PD-L1 (ab205921, Abcam, Cambridge, United Kingdom), and PD-1 (Roche). One pathologist blinded to the clinicopathologic factors evaluated the samples under a light microscope.

PD-1 and CD8 levels on the surface of lymphocytes were detected in the microenvironment of the tumor. PD-L1 expression was detected on the tumor cell plasma membrane or in the cytoplasm.

### IHC of PD-1, PD-L1, and CD8

For PD-1, tumors with staining in ≥ 5% of the tumor-infiltrating lymphocytes were considered positive. For PD-L1, tumors with staining in ≥ 5% of the tumor cells (membranous and cytoplasmic staining) were considered positive.

The density of tumor-infiltrating lymphocytes was stratified into two categories by CD8: 0; undetectable, 1 + ; low density (0–30%), 2 + ; moderate–high density (≥ 30%). Cases that were 2 + were counted as positive in our analysis.

### Statistical analyses

Analysis of the correlation between PD-1, PD-L1, and CD8 expression and clinicopathological characteristics was performed using the chi-squared test. Furthermore, correlations between the expression levels of each of these three proteins were determined using the chi-squared test. The progression-free survival (PFS) and overall survival (OS) rates were analyzed with the Kaplan–Meier method using the log-rank test.

Univariate and multivariate Cox proportional hazard regression models were used with binomial logistic regression for ordered categorical variables. Statistical calculations were performed using IBM SPSS (IBM, Armonk, NY, USA), version 23. A *P* value < 0.05 was considered statistically significant.

## Results

### Clinical and pathological features

The patients’ clinicopathological characteristics are summarized in Table 1. In the present study, the clinical stages were determined according to the guidelines of the International Federation of Gynecology and Obstetrics (FIGO): stages IA, IB1, IB2, IIA, IIB, IIIB, and IVB were observed in 5, 36, 12, 5, 14, 8, and 2 cases, respectively. The patients were initially treated as follows: 72 patients underwent radical hysterectomy followed by adjuvant therapy such as concurrent chemoradiotherapy (CCRT). In Japan, radical hysterectomy has always been considered the standard treatment strategy for locally advanced cases, such as cases of stage 1B2, 2A, and 2B disease. Radical hysterectomy was performed for all patients with a surgical resection margin of 1–2 cm. The surgical margin was confirmed in all patients, and the number of isolated lymph nodes (LNs) was more than 20. Nine patients with advanced stage cancer were initially treated with CCRT. One patient underwent chemotherapy without surgery because of multiple distant metastases. Radiotherapy (whole pelvic irradiation) or chemotherapy (paclitaxel 175 mg/m^2^ and carboplatin area under the curve = 5 mg/m^2^) was performed postoperatively in patients with a high recurrence risk (locally advanced stage, non-SCC histology type, bulky tumor (≥ 4 cm), deep infiltration depth of the cervical tumor (grade 2 or 3), lymph node metastasis, or lymphovascular space invasion).

**Table 1:** Characteristics of the patients with cervical adenocarcinoma

Number of patients	*n* = 82
Age (years) median (range)	50.4 (30–85)
FIGO stage *n*, (%)	
IA	5 (6.1)
IB	48 (58.5)
IIA	5 (6.1)
IIB	14 (17.1)
IIIA	0
IIIB	8 (9.8)
IVA	0
IVB	2 (2.4)
Tumor size (cm) median (range)	32.7 (0–80)
Unknown	5
Tumor size	
< 4 cm	49 (59.6)
≥ 4 cm	33 (40.4)
LVI	
Yes	39 (47.6)
No	29 (35.4)
Unknown	14 (17.0)
Metastatic paraaortic LN	
Yes	17 (20.7)
No	65 (79.3)
Metastatic pelvic LN	
Yes	3 (3.7)
No	79 (92.3)
Metastasis distance	
Yes	1 (1.2)
No	81 (98.8)
Treatment	
Operation only	24 (29.2)
Operation + adjuvant (RT or CCRT or CT)	48 (58.5)
Radiotherapy (RT, CCRT)	8 (9.8)
Chemotherapy	2 (2.5)
Recurrence within 5 years	
Yes	20 (24.4)
No	60 (75.6)
Death within 5 years	
Yes	19 (23.2)
No	63 (76.8)

*LVI* lymphovascular invasion, *RT* radiotherapy, *CCRT* concurrent chemoradiotherapy, *CT* chemotherapy

The chemotherapy regimens adopted were paclitaxel plus carboplatin, paclitaxel plus cisplatin, docetaxel plus carboplatin, irinotecan plus cisplatin, and gemcitabine. In the CCRT regimen, cisplatin was administered weekly in 5–6 courses of 40 mg/m^2^.

**Fig. 1:**
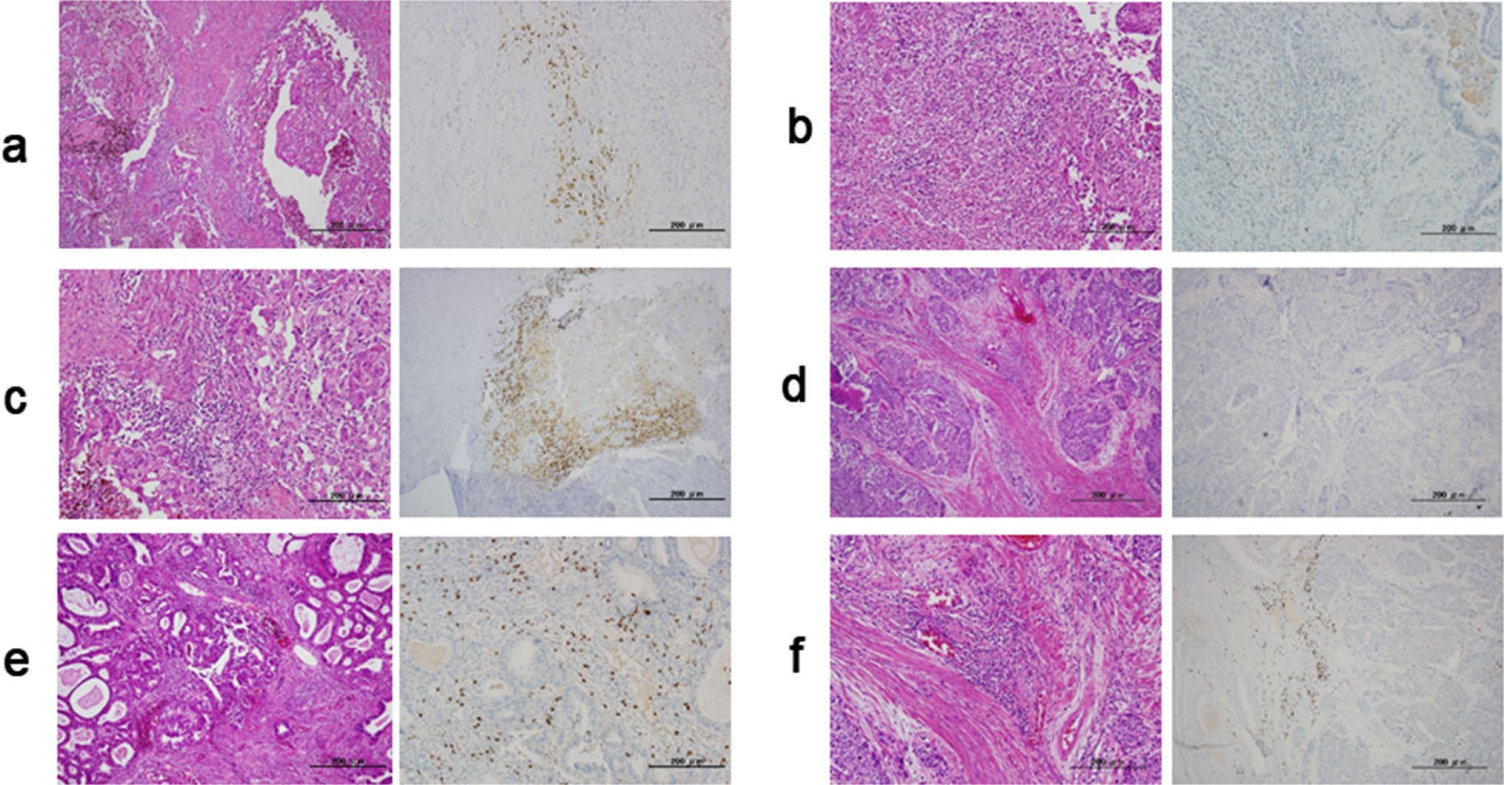
**a**–**f** HE staining and immunohistochemical analysis of the specimens from patients with cervical adenocarcinoma. **a**, **b** Immunostaining of PD-1. A, positive expression of PD-1; B, no expression of PD-1. **c**, **d** Immunostaining of PD-L1. C, positive expression of PD-L1; D, no expression of PD-L1. **e**, **f** Immunostaining of CD8: E, CD8 expression score of + 2; F, CD8 expression scores of 0 and + 1

Figure 1 shows representative cases that were positive or negative for PD-1, PD-L1, and CD8 expression.

### Correlation between the expression levels of PD-1, PD-L1, and CD8 in cervical adenocarcinoma

We observed a significant inverse correlation between PD-1 expression and CD8 expression on the tumor-infiltrating lymphocytes (*p* = 0.001, chi-square test; Table 2a). We also found a significant inverse correlation between PD-L1 expression and CD8 expression in the tumor-infiltrating lymphocytes (*p* = 0.027, chi-square test; Table 2b). Furthermore, PD-1 expression was significantly positively correlated with PD-L1 expression in the tumor-infiltrating lymphocytes (*p* = 0.029, chi-square test; Table 2c).

**Table 2:** Relationship between PD-1 expression and CD8 expression

2a			
Parameter	PD-1 positive	PD-1 negative	*p* value
	*n* = 23	*n* = 59	
CD8 *n*, %			0.001
Positive	9 (39.1)	46 (78.0)	
Negative	14 (60.9)	13 (22.0)	

2b. Relationship between PD-L1 expression and CD8 expression			
Parameter	PD-1 positive	PD-1 negative	*p* value
	*n * = 16	*n * = 66	
CD8 *n*, %			0.027
Positive	7 (43.8)	48 (72.7)	
Negative	9 (56.7)	18 (27.3)	

2c Relationship between PD-1 expression and PD-L1 expression			
Parameter	PD-1 positive	PD-1 negative	*p* value
	*n * = 16	*n * = 66	
PD-1 *n*, %			0.029
Positive	8 (50.0)	15 (22.7)	
Negative	8 (50.0)	51 (77.3)	

There was no significant correlation between the expression levels of PD-1, PD-L1, and CD8 and the clinicopathological factors examined (Supplementary Table 1a–c).

### Correlation between the prognosis of cervical adenocarcinoma and the expression levels of PD-1, PD-L1, and CD8

**Fig. 2:**
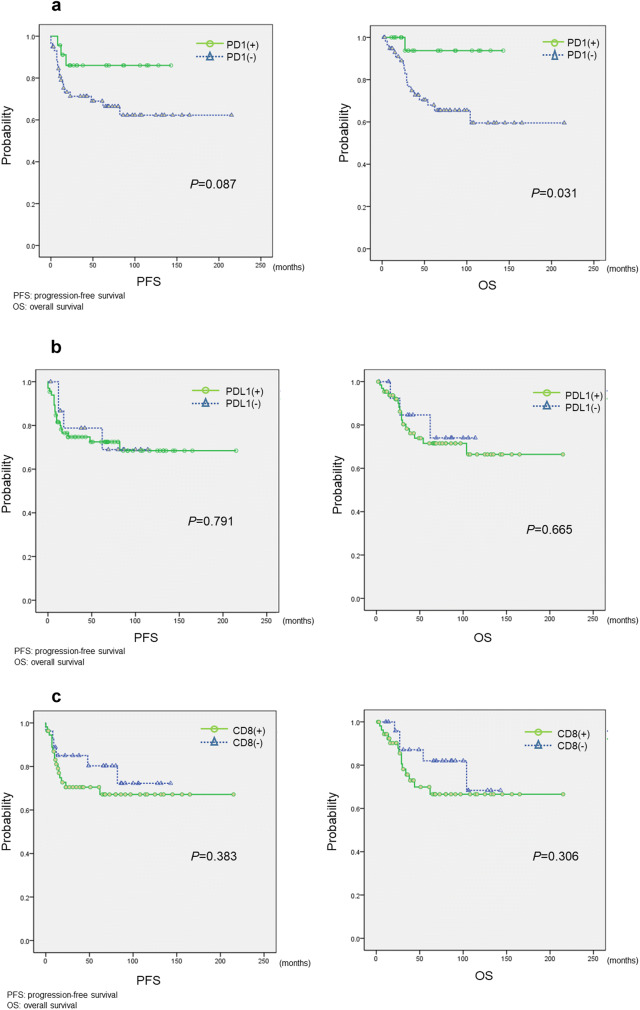
**a**–**c** Kaplan–Meier analysis of progression-free and overall survival associated with each immune-checkpoint-related molecular. **a** Kaplan–Meier analysis of progression-free survival and overall survival between the PD-1(+) group and PD-1(−) group. **b** Kaplan–Meier analysis of progression-free survival and overall survival between the PD-L1( +) group and PD-L1(−) group. **c** Kaplan–Meier analysis of the progression-free survival and overall survival between the CD8(+) group and CD8(−) group

Cervical adenocarcinoma patients with PD-1 lymphocyte positivity had worse overall survival (*p* = 0.031, log-rank test, Fig. 2a) as well as worse progression-free survival (*p* = 0.087, log-rank test, Fig. 2a) compared with patients with PD-1 expression negativity. Cervical adenocarcinoma patients with PD-L1 positivity did not have a significantly worse progression-free survival and overall survival (*p* = 0.791, log-rank test. *p* = 0.665, log-rank test, Fig. 2b) compared with adenocarcinoma patients with PD-L1 negativity (Fig. 2b). Cervical adenocarcinoma patients with CD8-positive lymphocytes also did not have a significantly worse progression-free survival and overall survival (*p* = 0.383, log-rank test. *p* = 0.306, log-rank test, Fig. 2c) compared with adenocarcinoma patients without CD8-positive lymphocytes (Fig. 2c).

### Univariate analysis of the prognostic factors in patients with cervical adenocarcinoma

There were no significant differences in the progression-free survival (PFS) or overall survival (OS) between the positive and negative PD-1 cases. Similarly, there were no differences in PFS and OS between positive or negative CD8 and PD-1 cases. Tumor size and metastatic invasion of the lymph-vascular space were the only parameters significantly correlated with PFS and OS of the patients (Tables 3, 4).

**Table 3:** Univariate and multivariate analyses of progression-free survival using a Cox proportional hazards model in patients with cervical adenocarcinoma

Factors	Patients	Univariate analysis			Mutivariate analysis		
	*n*	HR	95% CI	*p* value	HR	95% CI	*p* value
Age (y)							
< 60	58	0.552	0.236–1.293	0.171			
≥ 60	24	Ref					
FIGO stage							
< IIB	58	0.194	0.081–0.464	< 0.0001	Ref		
≥ IIB	24	Ref			1.101	0.320–3.787	0.879
Histology							
Non-gastric type	69	0.819	0.277–2.422	0.819			
Gastric type	13						
Tumor size (mm)							
< 40	50	0.135	0.050–0.369	0.0001	0.18	0.046–0.702	0.013
≥ 40	32	Ref			Ref		
Metastasis pelvic lymph node							
Negative	65	0.379	0.162-.0889	0.026	0.583	0.147–2.307	0.442
Positive	17	Ref			Ref		
Metastasis paraaortic lymph node							
Negative	79	0.209	0.061–0.707	0.012	0.235	0.012–4.687	0.343
Positive	3	Ref			Ref		
Metastasis distance							
Negative	81	0.078	0.009–0.644	0.018	0.454	0.042–4.941	0.517
Positive	1	Ref			Ref		
Metastasis LVI							
Negative	29	0.453	0.087–2.353	0.004	0.035	0.003–0.387	0.005
Positive	39	Ref			Ref		
PD-1							
Negative	59	Ref			Ref		
Positive	23	2.756	0.815–9.319	0.103	1.791	0.408–7.854	0.44
PD-L1							
Negative	66	Ref			Ref		
Positive	16	1.153	0.391–3.420	0.792	6.251	0.610–64.077	0.123
CD8							
Negative	27	Ref			0.507	0.118–2.170	0.36
Positive	55	1.512	0.591–3.872	0.388	Ref

**Table 4:** Univariate and multivariate analyses of overall survival using a Cox proportional hazards model in patients with cervical adenocarcinoma

Factors	Patients	Univariate analysis			Mutivariate analysis		
	*n*	HR	95% CI	*p* value	HR	95% CI	*p* value
Age (y)							
< 60	58	0.58	0.233–1.446	0.243			
≥ 60	24	Ref					
FIGO stage							
< IIB	58	0.222	0.087–0.565	0.002	0.968	0.284–3030	0.958
≥ IIB	24	Ref			Ref		
Histology							
Non-gastric type	69	0.67	0.222–2.021	0.477			
Gastric type	13	Ref					
Tumor size (mm)							
< 40	50	0.168	0.060–0.468	0.001	0.171	0.036–0.812	0.026
≥ 40	32	Ref			Ref		
Metastasis pelvic lymphnode							
Negative	65	0.515	0.202–1.309	0.163			
Positive	17	Ref					
Metastasis paraaortic lymphnode							
Negative	79	0.321	0.074–1.405	0.131			
Positive	3	Ref					
Metastasis distance							
Negative	81	0.026	0.002–0.283	0.003	0.102	0.009–1.205	0.07
Positive	1	Ref			Ref		
Metastasis LVI							
Negative	29	0.053	0.007–0.416	0.005	0.034	0.002–0.521	0.015
Positive	39	Ref			Ref		
PD-1							
Negative	59	Ref			Ref		
Positive	23	6.765	0.903–50.705	0.063	5.311	0.549–51.377	0.149
PD-L1							
Negative	66	Ref			Ref		
Positive	16	1.331	0.382–2.196	0.667	2.414	0.439–13.264	0.311
CD8							
Negative	27	Ref			0.286	0.055–1.498	0.139
Positive	55	1.694	0.609–4.710	0.313	Ref		

## Discussion

The present study reports two major findings: First, we revealed a significant inverse correlation between PD-1 and CD8 expression levels of tumor-infiltrating lymphocytes. We also found a significant inverse correlation between PD-L1 expression and CD8 expression on tumor-infiltrating lymphocytes. These findings have already been demonstrated in previous studies on ovarian cancer [6] and other malignant tumors [7, 8]. In cervical adenocarcinoma, tumor cells expressing PD-L1 may be protected from the destructive activity of CD8 + lymphocytes. The reduction of CD8 expression levels may not be the only mechanism by which PD-L1 promotes tumor immune escape. It may be possible that PD-L1 on tumor cells induces functional impairment of tumor-specific T cells without reducing their CD8 levels, as reported for antiviral T cells [9, 10]. A recent meta-analysis study concluded that the correlations between the survival of cancer patients and the expression of PD-L1 vary among different tumor types [11].

Second, we showed that the high expression level of PD-1 is associated with a poor prognosis in cervical adenocarcinoma patients (Fig. 2a). Some previous studies showed no significant difference between the expression levels of immune-checkpoint associated proteins and the prognosis of cervical adenocarcinoma patients [12]. However, the results of these studies were controversial [13]. In the current study, the level of CD8 on tumor-infiltrating lymphocytes was not found to be associated with a favorable prognosis. A similar result was reported in a previous study on cervical squamous cell carcinoma and adenocarcinoma [14–16].

We also observed a significant correlation between high PD-1 expression levels and worse overall survival of cervical adenocarcinoma patients. The expression levels of PD-L1 and CD8 were not significantly correlated with the patients’ prognosis. The overexpression of PD-L1 on tumor cells might inhibit the activity of CD8-expressing lymphocytes in the microenvironment of the tumor. Previous studies suggested that CD8 + lymphocytes could not engage with PD-L1-positive tumor cells. Therefore, the expression levels CD8 and PD-L1 are expected to be inversely correlated [6, 8].

If we use PD-1 inhibitor for cervical adenocarcinoma patients with high PD-1 expression, their immune activity against tumor cells would recover and may decrease the size of the tumor. Future research on biomarkers that predict response to checkpoint blockade immunotherapies could increase their durable responses. It is known that PD-1 in combination with PD-L1 and PD-L2 decreases the activity of the CD8-positive killer T cells. Therefore, the diminished T cell activity may be recovered by inhibiting the PD-1 signal using the anti-PD-1 antibody, and the function of the CD8-positive cell will be restored. In the current study, the expression of PD-1, PD-L1 and CD8 may serve as a marker for the use of immune checkpoint inhibitors as the initial treatment [17, 18].

The combination therapy with anti-cytotoxic T-lymphocyte-associated antigen-4 (CTLA-4) antibody and anti-PD-1 antibody was approved for use for unresectable malignant melanoma by the FDA in America in 2015 [19, 20]. In addition, it was noted that the regulatory T cells (Treg cells) and immunosuppressive cells, such as myeloid-derived suppressor cells (MDSCs), were decreased with a certain anticancer agent. Cyclophosphamide (CP) and 5-FU are among the representative anticancer drugs that decrease the Treg cell activity [21, 22]. Gemcitabine (GEM) and docetaxel (DOC) are also known to control the Treg cell activity [23]. I Chemotherapeutic agents, such as 5-FU and gemcitabine, may be used during the initial treatment in combination with the immune checkpoint inhibitors due to the associated immunomodulatory effects in patients with low CD8 expression because 5-FU and gemcitabine increase the efficacy of the activity of the CD8-positive T cells through their cytotoxic effect [24–26]. Furthermore, vascular endothelial growth factor (VEGF) is known for its growth-promoting function with respect to the regulatory Treg cells. Therefore, the treatment that targeted VEGF and VEGF receptor (VEGFR) could inhibit the Treg activity and help augment the effect of tumor immunity. For example, the anti-VEGFR2 antibody could control the increase in the Treg activity [27, 28]. Additionally, the combination therapy with anti-VEGF antibody and with immune checkpoint inhibitors was evaluated in clinical trial, its positive effects have been identified [29–32]. In the previous report, we also suggested the use of combination therapy with immune checkpoint inhibitors and conventional chemotherapeutic agents, like VEGF inhibitors, and poly ADP-ribose polymerase (PARP) inhibitors [33].

There are some limitations to our study. The first limitation is that we investigated the expression levels of PD-1 and CD8 on lymphocytes only using immunohistochemical analysis. Although previous studies also used this strategy, it was difficult to determine the expression levels when there were only a few lymphocytes in the tumor microenvironment. For example, unlike that in endometrial carcinoma, the number of tumor-infiltrating lymphocytes is small in gynecological malignant tumors, such as ovarian carcinoma and cervical carcinoma. Thus, it was difficult to determine the expression levels of proteins in these lymphocytes.

Another limitation was that we did not count lymphocytes separately in the tumor epithelium and tumor stroma as was done in previous studies [12, 13]. As we speculated that the function of tumor-infiltrating lymphocytes is not different in the epithelium and stroma, in the current study, we counted together the lymphocytes in both sections. In future studies, it is necessary to count lymphocytes individually in the stroma and epithelial sections.

Furthermore, the use of PD-L2 antibodies has recently been suggested as a new strategy of immune-checkpoint therapy [34]. The expression level of PD-L2 has already been examined in some tumors. Furthermore, some studies demonstrated that anti-PD-L1 plus anti-PD-L2 therapy has improved antitumor effects [35].

Finally, as many cervical adenocarcinoma patients still have a poor prognosis, it is essential to develop new immunotherapies for the treatment of this cancer.

## Electronic supplementary material

Below is the link to the electronic supplementary material.

Supplementary file1 Supplementary Table 1 a. b. c. Relationship between the patients’ clinicopathological factors and immune-checkpoint-related molecules according to the Chi-square test. a: Relationship between the patients’ clinicopathological factors and PD-1. (XLSX 13 kb)

Supplementary file2 b: Relationship between the patients’ clinicopathological factors and PD-L1. (XLSX 13 kb)

Supplementary file3 c: Relationship between the patients’ clinicopathological factors and CD8. There was no significant correlation between these factors and immune-checkpoint-related molecules. (XLSX 13 kb)

## Abbreviations

PD-1: Programmed cell death-1
PD-L1: Programmed cell death-ligand 1
CA: Cervical adenocarcinoma
ICI: Immune-checkpoint inhibition
LVI: Lymphovascular invasion
RT: Radiotherapy
CT: Chemotherapy
CTLA-4: Cytotoxic T-lymphocyte-associated antigen-4
Treg cell: Regulatory T cell
MDSCs: Myeloid-derived suppressor cells
CP: Cyclophosphamide
GEM: Gemcitabine
DOC: Docetaxel
VEGF: Vascular endothelial growth factor
PARP: Poly-ADP-ribose polymerase

## References

[1] Vital Statistics Japan (Ministry of Health, Labour and Welfare, Cancer Registry and Statistics. Cancer Information Service, National Cancer Center, Japan

[2] Hori M, Matsuda T, Shibata A, Katanoda K, Sobue T, Nishimoto H (2015). Cancer incidence and incidence rates in Japan in 2009: a study of 32 population-based cancer registries for the Monitoring of Cancer Incidence in Japan (MCIJ) project. Jpn J Clin Oncol.

[3] Vinh-Hung V, Bourgain C, Vlastos G, Cserni G, De Ridder M, Storme G, Vlastos AT (2007). Prognostic value of histopathology and trends in cervical cancer: a SEER population study. BMC Cancer.

[4] Wilbur DC, Colgan TJ, Ferenczy AS, Kurman RJ, Carcangiu ML, Herrington CS (2014). Glandular tumors and precursors. World Health Organization Classification of Tumours Pathology and Genetics. Tumours Female Reprod Organs.

[5] Japanese Journal of Gynecologic Oncology, Statement for the Year 2012. 5-year results. (2019) Acta Obstetricaet Gynecologica Japonica: 727–747.

[6] Hamanishi J, Mandai M, Iwasaki M, Okazaki T, Tanaka Y, Yamaguchi K, Higuchi T, Yagi H, Takakura K, Minato N, Honjo T, Fujii S (2007). Programmed cell death 1 ligand 1 and tumor-infiltrating CD8+ T lymphocytes are prognostic factors of human ovarian cancer. Proc Natl Acad Sci U S A.

[7] Dong H, Strome SE, Salomao DR, Tamura H, Hirano F, Flies DB, Roche PC, Lu J, Zhu G, Tamada K, Lennon VA, Celis E, Chen L (2002). Tumor-associated B7–H1 promotes T-cell apoptosis: a potential mechanism of immune evasion. Nat Med.

[8] Iwai Y, Ishida M, Tanaka Y, Okazaki T, Honjo T, Minato N (2002). Involvement of PD-L1 on tumor cells in the escape from host immune system and tumor immunotherapy by PD-L1 blockade. Proc Natl Acad Sci U S A.

[9] Barber DL, Wherry EJ, Masopust D, Zhu B, Allison JP, Sharpe AH, Freeman GJ, Ahmed R (2006). Restoring function in exhausted CD8 T cells during chronic viral infection. Nature.

[10] Hirano F, Kaneko K, Tamura H, Dong H, Wang S, Ichikawa M, Rietz C, Flies DB, Lau JS, Zhu G, Tamada K, Chen L (2005). Blockade of B7–H1 and PD-1 by monoclonal antibodies potentiates cancer therapeutic immunity. Cancer Res.

[11] Wu P, Wu D, Li L, Chai Y, Huang J (2015). PD-L1 and Survival in Solid Tumors: a meta-analysis. PLoS ONE.

[12] Kawachi A, Yoshida H, Kitano S, Ino Y, Kato T, Hiraoka N (2018). Tumor-associated CD204_+_ M2 macrophages are unfavorable prognostic indicators in uterine cervical adenocarcinoma. Cancer Sci.

[13] Heeren AM, Punt S, Bleeker MC, Gaarenstroom KN, van der Velden J, Kenter GG, de Gruijl TD, Jordanova ES (2016). Prognostic effect of different PD-1 expression patterns in squamous cell carcinoma and adenocarcinoma of the cervix. Mod Pathol.

[14] Punt S, van Vliet ME, Spaans VM, de Kroon CD, Fleuren GJ, Gorter A, Jordanova ES (2015). FoxP3(+) and IL-17(+) cells are correlated with improved prognosis in cervical adenocarcinoma. Cancer Immunol Immunother.

[15] Karim R, Jordanova ES, Piersma SJ, Kenter GG, Chen L, Boer JM, Melief CJ, van der Burg SH (2009). Tumor-expressed B7–H1 and B7-DC in relation to PD-1+ T-cell infiltration and survival of patients with cervical carcinoma. Clin Cancer Res.

[16] Spaans VM, Peters AA, Fleuren GJ, Jordanova ES (2012). HLA-E expression in cervical adenocarcinomas: association with improved long-term survival. J Transl Med.

[17] Okazaki T, Chikuma S, Iwai Y, Fagarasan S, Honjo T (2013). A rheostat for immune responses: the unique properties of PD-1 and their advantages for clinical application. Nat Immunol.

[18] Lorenz U (2009). SHP-1 and SHP-2 in T cells: two phosphatases functioning at many levels. Immunol Rev.

[19] Hodi FS, Chiarion-Sileni V, Gonzalez R, Grob JJ, Rutkowski P, Cowey CL, Lao CD, Schadendorf D, Wagstaff J, Dummer R, Ferrucci PF, Smylie M, Hill A, Hogg D, Marquez-Rodas I, Jiang J, Rizzo J, Larkin J, Wolchok JD (2018). Nivolumab plus ipilimumab or nivolumab alone versus ipilimumab alone in advanced melanoma (CheckMate 067): 4-year outcomes of a multicentre, randomised, phase 3 trial. Lancet Oncol.

[20] Larkin J, Hodi FS, Wolchok JD, Larkin J (2015). Combined nivolumab and ipilimumab or monotherapy in untreated melanoma. N Engl J Med.

[21] Sistigu A, Viaud S, Chaput N, Bracci L, Proietti E, Zitvogel L (2011). Immunomodulatory effects of cyclophosphamide and implementations for vaccine design. Semin Immunopathol.

[22] Shevchenko I, Karakhanova S, Soltek S, Link J, Bayry J, Werner J, Umansky V, Bazhin AV (2013). Low-dose gemcitabine depletes regulatory T cells and improves survival in the orthotopic Panc02 model of pancreatic cancer. Int J Cancer.

[23] Suzuki E, Kapoor V, Jassar AS, Kaiser LR, Albelda SM (2005). Gemcitabine selectively eliminates splenic Gr-1+/CD11b+ myeloid suppressor cells in tumor-bearing animals and enhances antitumor immune activity. Clin Cancer Res.

[24] Vincent J, Mignot G, Chalmin F, Ladoire S, Bruchard M, Chevriaux A, Martin F, Apetoh L, Rébé C, Ghiringhelli F (2010). 5-Fluorouracil selectively kills tumor-associated myeloid-derived suppressor cells resulting in enhanced T cell-dependent antitumor immunity. Cancer Res.

[25] Davis M, Conlon K, Bohac GC, Barcenas J, Leslie W, Watkins L, Lamzabi I, Deng Y, Li Y, Plate JM (2012). Effect of pemetrexed on innate immune killer cells and adaptive immune T cells in subjects with adenocarcinoma of the pancreas. J Immunother.

[26] Peng J, Hamanishi J, Matsumura N, Abiko K, Murat K, Baba T, Yamaguchi K, Horikawa N, Hosoe Y, Murphy SK, Konishi I, Mandai M (2015). chemotherapy induces programmed cell death-ligand 1 overexpression via the nuclear factor-κb to foster an immunosuppressive tumor microenvironment in ovarian cancer. Cancer Res.

[27] Terme M, Pernot S, Marcheteau E, Sandoval F, Benhamouda N, Colussi O, Dubreuil O, Carpentier AF, Tartour E, Taieb J (2013). VEGFA-VEGFR pathway blockade inhibits tumor-induced regulatory T-cell proliferation in colorectal cancer. Cancer Res.

[28] Ott PA, Hodi FS, Buchbinder EI (2015). Inhibition of immune checkpoints and vascular endothelial growth factor as combination therapy for metastatic melanoma: an overview of rationale, preclinical evidence, and initial clinical data. Front Oncol.

[29] Wada J, Suzuki H, Fuchino R, Yamasaki A, Nagai S, Yanai K, Koga K, Nakamura M, Tanaka M, Morisaki T, Katano M, Wada J (2009). The contribution of vascular endothelial growth factor to the induction of regulatory T-cells in malignant effusions. Anticancer Res.

[30] Osada T, Chong G, Tansik R, Hong T, Spector N, Kumar R, Hurwitz HI, Dev I, Nixon AB, Lyerly HK, Clay T, Osada MMAT (2008). The effect of anti-VEGF therapy on immature myeloid cell and dendritic cells in cancer patients. Cancer Immunol Immunother.

[31] Manzoni M, Rovati B, Ronzoni M, Loupakis F, Mariucci S, Ricci V, Gattoni E, Salvatore L, Tinelli C, Villa E, Manzoni DM (2010). Immunological effects of bevacizumab-based treatment in metastatic colorectal cancer. Oncology.

[32] Shrimali RK, Yu Z, Theoret MR, Chinnasamy D, Restifo NP, Rosenberg SA, Shrimali RK (2010). Antiangiogenic agents can increase lymphocyte infiltration into tumor and enhance the effectiveness of adoptive immunotherapy of cancer. Cancer Res.

[33] Yamashita H, Nakayama K, Ishikawa M, Ishibashi T, Nakamura K, Sawada K, Yoshimura Y, Tatsumi N, Kurose S, Minamoto T, Iida K, Razia S, Ishikawa N, Kyo S (2019). Relationship between microsatellite instability, immune cells infiltration, and expression of immune checkpoint molecules in ovarian carcinoma: immunotherapeutic strategies for the future. Int J Mol Sci.

[34] Yearley JH, Gibson C, Yu N, Moon C, Murphy E, Juco J, Lunceford J, Cheng J, Chow LQM, Seiwert TY, Handa M, Tomassini JE, McClanahan T (2017). PD-L2 expression in human tumors: relevance to anti-PD-1 therapy in cancer. Clin Cancer Res.

[35] Umezu D, Okada N, Sakoda Y, Adachi K, Ojima T, Yamaue H, Eto M, Tamada K (2019). Inhibitoryfunctions of PD-L1 and PD-L2 in the regulation of anti-tumor immunity in murine tumor microenvironment. Cancer Immunol Immunother.

